# Evaluation of baseline F-18 FDG positron emission tomography in the diagnosis and assessment of giant cell arteritis

**DOI:** 10.55730/1300-0144.5767

**Published:** 2024-01-05

**Authors:** Burak İNCE, Emine Göknur IŞIK, Zeynep Gözde ÖZKAN, Yasemin YALÇINKAYA, Bahar ARTIM ESEN, Ahmet GÜL, Murat İNANÇ

**Affiliations:** 1 Division of Rheumatology, Department of Internal Medicine, İstanbul Faculty of Medicine, İstanbul University, İstanbul, Turkiye; 2 Department of Nuclear Medicine, İstanbul Faculty of Medicine, İstanbul University, İstanbul, Turkiye

**Keywords:** Giant cell arteritis, large vessel vasculitis, positron emission tomography

## Abstract

**Background/aim:**

The aim of this study is to evaluate the baseline F18-FDG PET/CT findings of individuals diagnosed with giant cell arteritis (GCA) and to explore its association with clinical findings and classification criteria.

**Materials and methods:**

We analysed data from patients who underwent F18-FDG PET/CT scans to investigate large vessel (LV) involvement between 2010 and 2019. Only patients with a clinical diagnosis of GCA and at least 6 months of follow-up were included. We compared initial clinical features and laboratory findings based on the presence of LV vasculitis on PET/CT and the maximum standard uptake value (SUVmax) of vascular territories.

**Results:**

Twenty-nine patients (median age at diagnosis: 70, F/M: 24/5) were included in the study. Among them, 21 patients (72.4%) presented with cranial symptoms, while 8 patients (27.5%) had isolated LV-GCA. Twenty-two patients (75.9%) met the ACR/EULAR 2022 GCA classification criteria. LV vasculitis was detected on PET/CT in 23 patients (79.3%). A positive correlation was observed between SUVmax in the thoracic aorta and both CRP and ESR levels (r = 0.50, p = 0.026 and r = 0.63, p = 0.002, respectively). PET/CT positive patients were found to be younger (p = 0.016) and more frequently female (p = 0.017). They also exhibited fewer headaches (56.5% vs. 100%, p = 0.04), experienced fewer flares during follow-up (p = 0.03), and had a lower cumulative glucocorticoid dose at the 6th month (p = 0.036). Comparison of PET/CT-positive patients (n = 23) based on the fulfilment of the ACR/EULAR 2022 classification criteria revealed that patients who met these criteria were older (p = 0.02) and had significantly lower CRP levels at diagnosis (p = 0.02).

**Conclusion:**

The performance of F18-FDG PET/CT in diagnosing LV involvement in GCA is favourable, and the severity of FDG uptake in the vessel wall correlates with the acute phase response. Patients with extracranial involvement on PET/CT exhibit distinct features, including a younger age and female predominance. Additionally, these patients appear to experience fewer relapses and require lower doses of glucocorticoids. However, the clinical significance of PET/CT in patients who met ACR/EULAR classification criteria, predominantly consisting of patients with ischemic cranial symptoms, could not be determined in our study.

## 1. Introduction

Giant cell arteritis (GCA) is a systemic granulomatous vasculitis affecting the aorta and its major branches [1]. It predominantly afflicts individuals over the age of 50, with a noted female predominance [2]. Traditionally, the diagnosis and classification of GCA relied heavily on temporal artery biopsy (TAB) examination, alongside clinical findings indicative of cranial artery ischemia, such as new-onset temporal headache, jaw claudication, and sudden vision loss [3]. However, the introduction of temporal artery ultrasound and other cross-sectional imaging techniques has notably enhanced diagnostic accuracy while reducing the necessity for TAB [4–6]. Recently, the ACR/EULAR 2022 Giant cell arteritis classification criteria, incorporating imaging methods were established [7]. Furthermore, examination of extracranial arteries by computed tomography (CT), positron emission tomography (PET) and magnetic resonance imaging (MRI) facilitated the identification of disease patterns without cranial manifestations defined as large vessel GCA (LV-GCA) [8]. Among these imaging techniques, F18-FDG PET/CT holds numerous advantages, including precise detection of LV inflammation, diagnosis of polymyalgia rheumatica (PMR), and differential diagnosis of vasculitis [9]. In this study, we aimed to evaluate the baseline F18-FDG PET/CT findings of patients with GCA in our single-centre cohort and investigate their association with clinical findings and the latest classification criteria.

## 2. Patients and method

In this retrospective study, we analysed data from patients who underwent PET/CT scans with a preliminary diagnosis of vasculitis and were followed up for at least 6 months with a diagnosis of GCA between 2010 and 2019.

Data were collected from patient records using a predefined protocol developed in accordance with current recommendations [10]. The study protocol encompassed demographic information, clinical features, and laboratory values during diagnosis, treatment details (including cumulative glucocorticoid (GC) dose at the 6th month and tocilizumab administration), and information regarding disease flares. For classification purposes, the ACR/EULAR 2022 classification criteria at the time of diagnosis and EULAR/ACR PMR Classification criteria were retrospectively applied. A flare was defined as the reappearance of clinical signs attributable to GCA, accompanied by an increase in acute phase reactants (erythrocyte sedimentation rate (ESR) ≥30 mm/h and/or mean C-reactive protein (CRP) ≥10 mg/L), which subsequently led to a treatment adjustment by the attending rheumatologist after remission had been achieved.

F18-FDG PET/CT scans were conducted within a maximum of 3 days after the initiation of glucocorticoid therapy in our patients. All patients adhered to a minimum 6-h fasting period before imaging, and their blood glucose levels were confirmed to be below 200 mg/dL at the time of tracer injection. PET/CT scans were started 60 min after the intravenous administration of 18F-FDG. Whole-body PET scans were performed using a Discovery IQ PET/CT scanner (GE Healthcare). Two experienced nuclear medicine experts, blinded to clinical and laboratory data, reevaluated all images. SUVmax (maximum standardized uptake value) was determined by drawing regions of interest (ROI) in the vascular structures and compared with the SUVmax of the mediastinal blood pool. Furthermore, patients with isolated aortitis on PET/CT scans underwent exclusion assessments for other potential causes of aortitis and periaortitis, such as infection, IgG4-related disease, or retroperitoneal fibrosis. Patients demonstrating increased FDG uptake on PET/CT scans were categorized as belonging to the PET/CT-positive group.

Statistical analyses were conducted using IBM SPSS Statistics for Windows, v. 25.0 (IBM Corp., Armonk, NY, USA). Continuous variables are presented as mean (SD) or median (range), while categorical variables are expressed as numbers and percentages. For categorical comparisons, the chi-squared test and logistic regression analysis were employed. T-test and Mann–Whitney U test were used for the comparison of continuous variables, depending on the normality of the data. A p-value below 0.05 was considered statistically significant. Correlation analysis between laboratory values and SUVmax in PET/CT scans was conducted using the Pearson correlation test.

Ethics board approval was obtained from the Istanbul University, Istanbul Faculty of Medicine Ethics Committee (No. 1494-2019). Patient consent was not obtained due to the retrospective nature of the study.

## 3. Results

Twenty-nine consecutive patients were enrolled in the study, all of whom were aged over 50 years and had an ESR >50 mm/h at the time of diagnosis. Among them, twenty-four patients (82.8%) were female. The median age at diagnosis was 70 years (range: 51–81), with a mean follow-up duration of 37.3 ± 25.9 months (range: 6–112). The baseline demographic and clinical characteristics of the patients are summarized in Table 1. Ischemic cranial symptoms were observed in twenty-one patients (72.4%), while eight patients (27.5%) presented with isolated LV-GCA. Twenty-two patients (75.9%) met the ACR/EULAR 2022 GCA classification criteria, with twenty of them exhibiting ischemic cranial symptoms. All patients received high-dose oral steroids (1 mg/kg); methotrexate was administered to twenty-two patients (75.9%), and tocilizumab was given to five patients (17.2%) during the follow-up period. Nine patients (31%) experienced flares during the follow-up period.

The evaluation of F18-FDG PET/CT scans revealed pathological FDG uptake in large vessels in 23 patients (79.3%). Table 2 presents the arteries with pathological FDG retention along with the mean SUVmax for each artery. Notably, no patients exhibited pathological FDG uptake in cranial arteries. In the correlation analysis of vascular SUVmax and acute phase reactants, a positive correlation was observed between SUVmax detected in the thoracic aorta and both ESR (p = 0.002; r = 0.63) and CRP (p = 0.026; r = 0.5). Similarly, a positive correlation was found between SUVmax in the abdominal aorta and only ESR (p < 0.001; r = 0.77).

Comparison of patients based on PET/CT results revealed that those with positive PET/CT findings were younger (median age at diagnosis 67 vs. 74, p = 0.016), more frequently female (91% vs. 50%, p = 0.017), experienced significantly fewer headaches (56.5% vs. 100%, p = 0.04), had a decreased number of flares during follow-up (21.7% vs. 66.7%, p = 0.03), and received a lower cumulative GC dose at the 6th month (3.4 ± 1.2 vs. 4.6 ± 0.9 g, p = 0.036). The frequency of PMR was similar between PET/CT-positive and -negative groups (11/23 (47.8%) vs. 3/6 (50%), p = 0.92). Multivariate analysis for predicting factors associated with PET-CT positivity did not yield significant results (Table 3).

Among patients who tested positive on PET/CT, fulfilment of ACR/EULAR 2022 CC (n = 16) was associated with older age, lower CRP values, and a more frequent involvement of axillary arteries (Table 4).

## 4. Discussion

In this study, we demonstrated a high incidence of large vessel involvement detected by F18-FDG PET/CT in patients initially diagnosed with GCA in our single-centre cohort. The positivity of PET/CT scans may aid in identifying a distinct subphenotype of patients based on age, sex, and clinical characteristics, potentially offering prognostic insights such as a lower incidence of flares and reduced cumulative GC doses.The diagnostic and prognostic significance of extracranial large vessel involvement in GCA is a developing area of interest [8, 11]. The role of F18-FDG PET-CT imaging for this purpose was shown in cohort studies and took its place in recently published classification criteria. [5, 12]. In our GCA patient cohort, PET/CT revealed large vessel vasculitis in a significant proportion (79%) of cases. A study comprising patients with biopsy-proven giant cell arteritis reported a PET-CT sensitivity of 80%, consistent with our findings. Although our rates of positive TAB and temporal artery ultrasound (TA US) were lower, the inclusion of patients with at least 6 months of follow-up ensured the accuracy of GCA diagnosis. TAB was performed in 12 patients (41.4%), with six (20.7%) showing GCA-compatible findings. The preference for TAB was relatively low, likely due to previous data indicating lower positivity rates in patients with predominant large vessel vasculitis, which constituted the majority of our cohort [13]. Similarly, TA US was conducted in only eight patients (27.6%), of whom two were positive, reflecting the absence of an experienced ultrasonographist in our centre.

There is a moderate sensitivity of ACR/EULAR 2022 classification criteria in our cohort; nearly 25% of our patients had isolated LV-GCA and did not meet criteria. The significance of PET-CT in diagnosing GCA patients with ambiguous vascular manifestations is becoming increasingly apparent. Gonzalez-Gay et al. reported eleven patients without evident vascular manifestations among their cohort of 210 patients with biopsy-proven GCA [14]. These patients presented with constitutional symptoms, fever, unexplained anaemia, and PMR. The identification of LVV in such cases relies on excluding of alternative diagnoses such as malignancy and chronic infections [15]. PET/CT has also proven beneficial in diagnosing LV-GCA in patients with findings suggestive of PMR in a study from the same group. In that study, 51 (60.8%) of 84 patients with persistent PMR symptoms were found to have LV-GCA in PET/CT [16]. We think that broader use of vascular imaging techniques should be encouraged in these patients.

The thoracic aorta was the most commonly affected artery (75%), with nearly half of the patients exhibiting involvement of the subclavian and carotid arteries. Previous research has demonstrated an association between aortic involvement and structural damage such as aortic stenosis and insufficiency [11], underscoring the importance of documenting disease extent at diagnosis for prognostic purposes. While our data does not include structural changes, patients in our cohort exhibit clinical differences according to PET/CT results. PET/CT-positive patients were younger, more frequently female, experienced less headache at presentation, had a decreased number of flares during follow-up, and received lower cumulative glucocorticoid doses at the 6th month. The lower frequency of headache is not unexpected, given the presence of isolated LV-GCA patients without ischemic cranial symptoms identified by PET/CT in our cohort. The reduced number of flares and lower cumulative steroid doses are likely indicative of a milder disease course in patients with extracranial involvement. These patients, characterized by younger age and predominantly female sex in our cohort, also demonstrated a lower rate of relapses at the time of diagnosis, as shown in a previous cohort study involving 87 GCA patients [17].

Conversely, a large cohort study reported that large vessel involvement, such as upper limb ischemia and inflammation of the axillary and brachial arteries, was associated with relapses in GCA patients [18]. Aortitis on imaging were similar across relapsing and nonrelapsing patients in this study. Additionally, Dumont et al.’s study, encompassing 326 patients, found relapses to be associated with LV involvement on imaging and a less frequent history of stroke. However, the incidence of LV involvement in PET/CT was lower in their study compared to ours (33% vs. 80%) [19]. This discrepancy in LV involvement rates may stem from differences in the definition of LV involvement. Our findings were solely based on PET/CT results obtained during early disease, and none of our patients underwent screening via MRI or CT-angiography. The presence of ischemic findings in relapsing patients in both studies, such as stroke or limb ischemia, suggests a more resistant disease pattern, consistent with our previous report [20]. Therefore, our current findings may only be applicable to patients screened using F18-FDG PET/CT.

In the PET/CT-positive group, patients who met the ACR/EULAR 2022 classification criteria, predominantly consisting of individuals with ischemic cranial symptoms, exhibited several distinguishing features. These patients were older, had lower CRP levels, and showed more frequent involvement of axillary arteries on PET/CT. In a historical study conducted before the widespread use of PET/CT for diagnosing GCA, Cid et al. reported that a high acute phase response was associated with a lower frequency of cranial symptoms, suggesting a different clinical phenotype [21]. Our data supports this observation and provides further insight into describing this subphenotype. Another notable finding in this subgroup is the selectivity of arterial involvement based on clinical findings and classification criteria. Bilateral involvement of axillary and subclavian arteries has been highlighted as an important feature of GCA patients in two large vascular imaging studies and is included in the classification criteria [22]. Remarkably, all patients with bilateral axillary involvement in our cohort exhibited ischemic cranial symptoms and met the classification criteria. Therefore, this finding may serve as an indicator of a higher disease extent and reflect classical GCA features.

We detected a correlation between higher SUVmax values in the thoracic aorta with both higher CRP and ESR, and in the abdominal aorta with higher ESR. This observation suggests that PET/CT accurately reflects systemic inflammation in large vessel vasculitis, a finding supported by similar results reported in cohort studies of treatment-naive GCA patients [23, 24]. However, the impact of more prominent arterial inflammation on the clinical course remains to be investigated. The main limitations of our study include the small sample size and the lack of histopathologic evidence for most of our patients. The retrospective design of the study may have resulted in missing information in patient data, and the limited number of patients included in the study may have influenced the results of multivariate analysis. However, the study benefited from assessment and follow-up by the same rheumatologists and data collection using a predefined protocol.

It is worth noting that most patients did not have histopathologic evidence of GCA. Additionally, we only included PET/CT scans performed at our centre and evaluated the images by the same investigators to minimise heterogeneity.

In conclusion, F18-FDG PET/CT demonstrates favourable performance in diagnosing large vessel involvement in GCA, with the severity of uptake in the vessel wall in PET/CT correlating with the acute phase response. Patients exhibiting extracranial involvement on PET/CT display distinct clinical features, including a younger age and female predominance, and appear to have a more favourable prognosis, characterized by fewer relapses and lower corticosteroid requirements, at least in the short term. Furthermore, the involvement of axillary arteries is associated with classical GCA clinic. These findings warrant validation in further prospective studies involving a larger number of patients.

## Figures and Tables

**Table 1:** Demographics and baseline features of patients with Giant cell arteritis. CRP: C-reactive protein, ESR: erythrocyte sedimentation rate.

	n = 29	%
**Age, med (IQR)**	70 (14)	
**Sex (female)**	24	82.8
**Ischemic cranial symptoms/findings**	21	72.4
**New temporal headache**	19	65.5
**Abnormality in temporal artery examination**	6	20.7
**Scalp tenderness**	8	27.6
**Jaw claudication**	13	44.8
**Sudden vision loss**	3	10.3
**Diplopia**	2	6.9
**Vertigo**	2	6.9
**Ischemic cerebrovascular event**	1	3.4
**Systemic/peripheral findings**	28	96.5
**Fatigue**	26	89.7
**Fever (patient reported)**	16	55.2
**Weight loss**	15	51.7
**Limb claudication**	2	6.9
**Vascular bruit**	1	3.4
**Polymyalgia rheumatica**	14	48.2
**Positive temporal artery biopsy**	6	20.7
**Positive temporal artery ultrasound**	2	6.9
**Patients met ACR/EULAR 2022 criteria**	22	75.9
**CRP at diagnosis, mean ± SD (range)**	107 ± 90.6 (16–302)	
**ESR at diagnosis, mean ± SD (range)**	100 ± 30.1 (67–140)	

**Table 2:** Involvement of large vessels in PET/CT and maximum standard uptake values (SUVmax).

	n (%)	SUVmax (mean ± SD)
**Thoracic aorta**	22 (75.8)	4.15 ± 1.3
**Abdominal aorta**	16 (55.2)	4.8 ± 1.7
**Brachiocephalic artery**	15 (51.7)	3.3 ± 1.6
**Subclavian artery (right)**	13 (44.8)	3.1 ± 1.8
**Subclavian artery (left)**	14 (48.3)	3.3 ± 1.8
**Carotid artery (right)**	13 (44.8)	3.5 ± 1.2
**Carotid artery (left)**	15 (51.7)	3.6 ± 1.4
**Axillary artery (right)**	7 (24.1)	4.5 ± 1.9
**Axillary artery (bilateral)**	6 (20.7)	3.9 ± 2
**Common iliac artery (any)**	12 (41.4)	3.1 ± 1.5

**Table 3:** Comparison of PET/CT-positive and -negative patients in all group and patients who met ACR/EULAR 2022 classification criteria. GC: glucocorticoid, TA: temporal artery.

	All groups				Patients who met ACR/EULAR 2022 CC			
	PET/CT (+) (n = 23)	PET/CT (−) (n = 6)	p	OR (%95 CI)	PET /CT (+) (n = 16)	PET/CT (−) (n = 6)	p	OR (%95 CI)
**Female sex**	**21**	**3**	**0.017**	**5.7 (1.2–27)**	**14**	**3**	**0.02**	**0.7 (0.05–0.94)**
**Age at diagnosis, med (IQR)**	**67 (12)**	**74 (12)**	**0.016**		70(12)	74 (12)	0.06	
**Headache**	**13**	**6**	**0.04**	**1.7 (1.2–2.5)**	14	6	0.51	
**Scalp/TA tenderness**	5	3	0.06		6	4	0.54	
**Jaw claudication**	9	4	0.22		9	4	0.77	
**Sudden vision loss**	1	2	0.1		1	2	0.11	
**PMR**	11	3 (50)	0.9		11	3	0.3	
**Fever**	10	3	0.6		6	3	0.67	
**CRP at diagnosis, mg/dL**	110.6 ± 96.4	95.2 ± 69.2	0.75		79.4 ± 74.6	95.2 ± 69.2	0.39	
**ESR at diagnosis, mm/h**	103.3 ± 24.8	105.7 ± 15.6	0.82		97.1 ± 25.2	105.7 ± 15.6	0.54	
**Cumulative GC dose at 6th month (g), mean ± SD**	**3.4 ± 1.2**	**4.6 ± 0.9**	**0.036**		3.7 ± 1	4.6 ± 0.9	0.1	
**Flares (ever)**	**5**	**4**	**0.03**	**7.2 (1.01–51)**	5	4	0.16	
**Tocilizumab treatment**	4	1	0.96		4	1	0.62	

**Bold** font indicates statistical significance at the p < 0.05 level.

**Table 4:** Comparison of PET/CT-positive patients who met and did not meet ACR/EULAR 2022 classification criteria. GC: glucocorticoid.

	PET/CT-positive patients (n = 23)			
	Patients who met the criteria (n = 16) (%)	Patients who did not meet the criteria (n = 7) (%)	p	OR
**Age at diagnosis, med (IQR)**	**70 (12)**	**66.5 (10)**	**0.02**	
**CRP at diagnosis, mg/dL, mean ± SD**	**79.5 ± 74.7**	**153.1 ± 108.7**	**0.02**	
**ESR at diagnosis, mm/h, mean ± SD**	97.1 ± 25.2	116.4 ± 19.2	0.09	
**Cumulative CS dose at 6th month (g)**	3.7 ± 1	3 ± 1.5	0.33	
**Flares (ever)**	5 (31.3)	0 (0)	0.06	
**Tocilizumab treatment**	4 (25)	0 (0)	0.1	
**Vessel involvement in PET/CT**				
**Brachiocephalic truncus**	10 (62.5)	5 (71.4)	0.47	
**Right subclavian**	9 (56.3)	4 (57.1)	0.73	
**Left subclavian**	10 (62.5)	4 (57.1)	0.65	
**Right carotid artery**	9 (56.3)	4 (57.1)	0.73	
**Left carotid artery**	10 (62.5)	5 (71.4)	0.47	
**Right axillary artery**	**7 (43.8)**	**0 (0)**	**0.036**	**1.78 (1.15–2.8)**
**Both axillary arteries**	6 (37.5)	0 (0)	0.06	
**Thoracic aorta SUVmax, mean ± SD**	3.9 ± 1.1	4.6 ± 1.3	0.36	
**Abdominal aorta SUVmax, mean ± SD**	4.5 ± 1.2	5.3 ± 1.8	0.27	

**Bold** font indicates statistical significance at the p < 0.05 level.
